# Massive Lingual and Sublingual Haematoma following Postextractive Flapless Implant Placement in the Anterior Mandible

**DOI:** 10.1155/2015/839098

**Published:** 2015-05-17

**Authors:** Luisa Limongelli, Angela Tempesta, Vito Crincoli, Gianfranco Favia

**Affiliations:** Department of Interdisciplinary Medicine, Complex Operating Unit of Odontostomatology, Aldo Moro University, Piazza Giulio Cesare 11, 70124 Bari, Italy

## Abstract

Dental implants placement in the anterior mandible with flap or flapless technique is a routine procedure and is considered to be safe. However, serious life-threatening complications may occur. We report the first case of massive lingual and sublingual haematoma following postextractive implant placement in the anterior mandible with flapless technique. A 45-year-old female patient underwent placement of four immediately postextractive implants in the anterior mandible using flapless technique. During the procedure, the patient referred intense acute pain and worsening sign of airway obstruction, dysphagia, dyspnea, and speech difficulties. Bimanual compression of the mouth floor, lingual surface of the mandible, and submental skin was maintained for approximately 25 minutes in order to stop the bleeding. Computerized tomography highlighted the massive lingual and sublingual haematoma. The symptoms and signs had almost completely resolved in the next 48 hours. The prevention of these complications is mandatory with clinical and CT analyses, in order to highlight mandibular atrophy and to select carefully the correct length and angulation of bone drilling and to keep more attention to the flapless technique considering the elevation of a lingual mucoperiosteal flap to access the mandibular contour intraoperatively and to protect the sublingual soft tissues and vasculature in high risk cases.

## 1. Introduction

Placement of dental implants in the anterior mandible with flap or flapless technique is a routine procedure and is considered to be safe [1]. However, in some situations, serious or even life-threatening complications may occur. To date, at least 18 cases of massive lingual and sublingual haematoma following implant placement have been published; in all cases, access to bone by mucoperiosteal flap was described and mainly was by postextractive procedures. We report the first case of massive lingual and sublingual haematoma following postextractive implant placement in the anterior mandible with flapless technique.

## 2. Case Presentation

**Figure 1:**
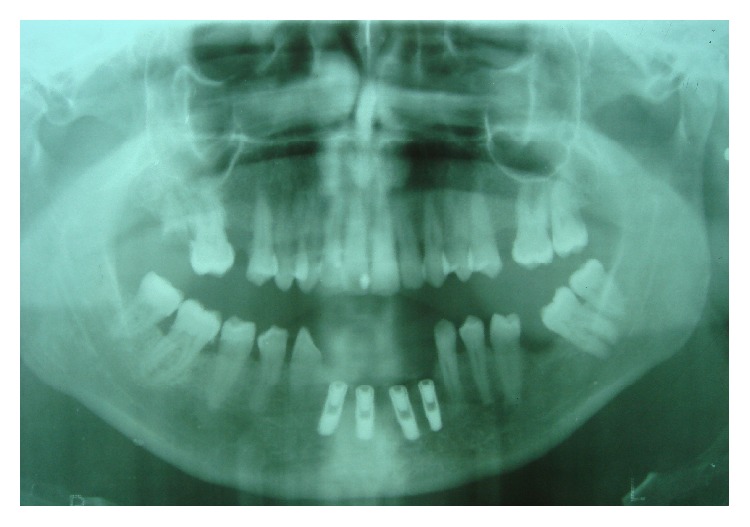
OPT after implant placement.

**Figure 2:**
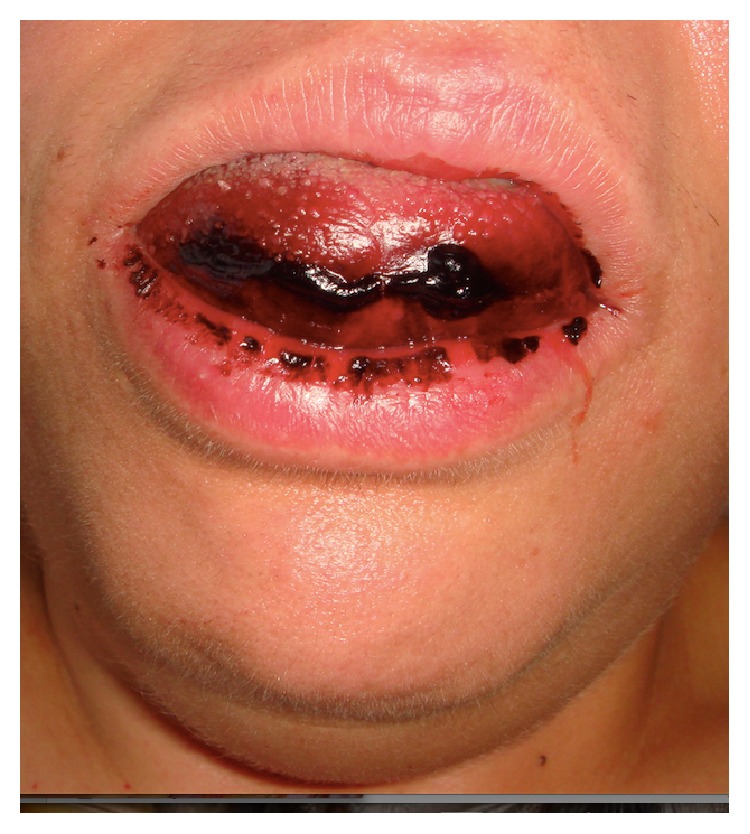
Clinical photograph of the extensive sublingual haematoma two hours after implant placement with the enlarged tongue (triple normal volume), displaced superiorly, pressed firmly against the palate, and protruding extraorally for 3 centimetres.

**Figure 3:**
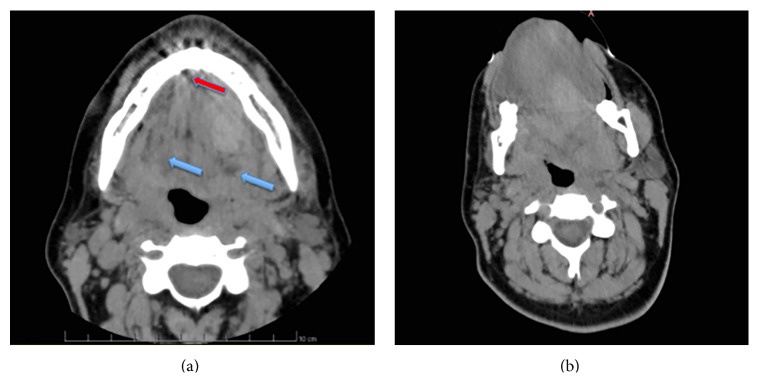
(a) Axial computed tomography image showing multiple areas of the whole mouth floor with different density (blue arrows) and the region of the cortical bone perforation (red arrow). (b) Axial computed tomography image showing the lingual, sublingual, and parapharyngeal haematoma with tongue displacement and restriction of the upper airways spaces.

**Figure 4:**
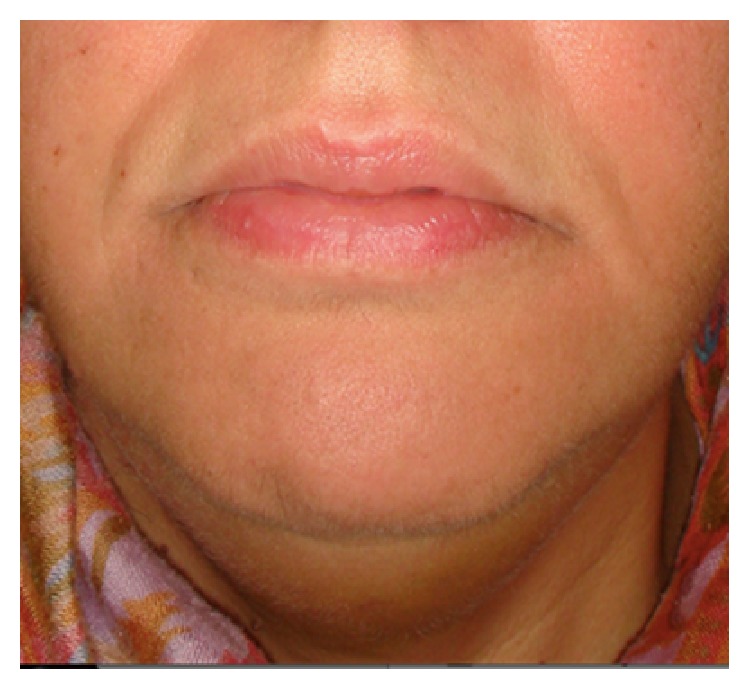
Clinical photograph 1 week later with good clinical recovery.

**Figure 5:**
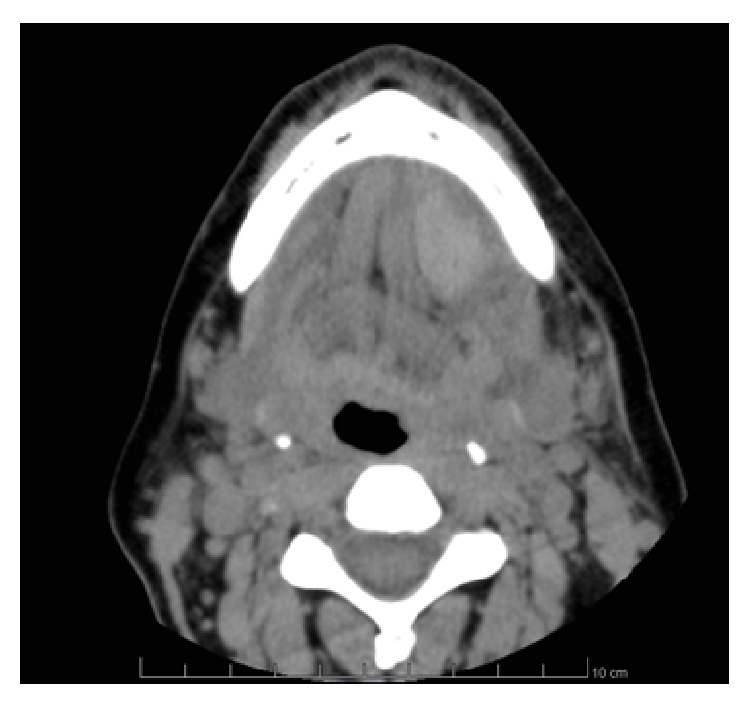
Axial computed tomography image showing almost complete regression of haematoma, normal dimension of upper airways spaces, and normal position of the tongue.

A 45-year-old female patient underwent placement of four immediately postextractive implants into the sockets of four parodontally compromised mandibular incisors using flapless technique (Figure 1). When deep drilling was performed in the left lateral incisor extraction socket, the patient referred intense acute pain; 15 minutes after the end of the surgical procedure, mouth floor and tongue swelling, stable pain, and worsening sign of airway obstruction with progressive dysphagia, dyspnea, and speech difficulties were noticed. Sixty minutes after, the dentist inserted the Guedel pattern airway and transferred the patient to the Department of Emergency and Health Urgent Care of the Policlinic of Bari. The clinical examination of oral cavity highlighted a red, indurated, and large swelling involving the sublingual, submental, and submandibular spaces bilaterally due to the haemorrhagic infarction of all soft tissues of the suprahyoid and parapharyngeal regions. The enlarged tongue (triple normal volume) was displaced superiorly, pressed firmly against the palate, and protruded extraorally for 3 centimetres (Figure 2). Bimanual compression of the mouth floor, lingual surface of the mandible, and submental skin was maintained for approximately 25 minutes in order to stop the bleeding. Enhanced multislice spiral computerized tomography with contrast medium was performed to analyse the bleeding focus and showed large and diffuse hyperdense ovoid areas of haemorrhagic origin involving the whole mouth floor, the left submandibular gland, and intrinsic and extrinsic tongue muscles probably depending on the breakage of sublingual artery (Figure 3). The vital signs were stable, her oxygen saturation was 96%, the respiratory rate was 17 breaths/minute, and she was tachycardic (115 beats/minute). Despite the massive mouth floor and tongue swelling, the patient was able to breathe autonomously so intubation or tracheostomy was not necessary. Intravenous access was secured and blood specimens were sent for haematological investigations and crossmatching. Therapies consisting in adrenaline, antibiotic prophylaxis, tranexamic acid, and steroids (hydrocortisone 1 g) were administered intravenously. The patient was monitored every 5 minutes, and no further enlargement of the large sublingual haematoma was observed; then, she was subsequently transferred for observation to the Intensive Care Unit. The symptoms and signs had almost completely resolved in the next 48 hours and the patient was discharged home on the fifth day with one week of oral treatment including amoxicillin-clavulanic acid (2 gr/die), ketoprofen (80 mg twice a day), and prednisone (30 mg/die) (Figures 4 and 5).

## 3. Discussion

The soft tissues of anterior floor of the mouth, delimited by mandibular arch, are supplied by a rich anastomosing vascular rete with three sources of arterial blood: (1) the submental arteries, (2) the sublingual arteries, and (3) the incisive arteries. The* submental artery*, the largest branch of facial artery, is given off from this one just as that vessel quits the submandibular gland: it runs forward upon the mylohyoideus, just below the body of the mandible, and beneath the digastricus. It supplies the surrounding muscles and anastomoses with the sublingual artery and with the mylohyoid branch of the inferior alveolar artery; at the symphysis menti, the submental artery turns upward over the inferior border of the mandible and divides into a superficial and a deep branch. The superficial branch passes between the integument and quadratus labii inferioris and anastomoses with the inferior labial artery; the deep branch runs between the muscle and the bone, supplies the lip, and anastomoses with the inferior labial and mental arteries. The* sublingual artery* arises at the anterior margin of the hyoglossus and runs forward between the genioglossus and mylohyoideus to the sublingual gland. It supplies the salivary glands and gives branches to the mylohyoideus and neighbouring muscles and to the mucous membrane of the mouth and gums. One branch runs behind the alveolar process of the mandible in the substance of the gum to anastomose with a similar artery from the other side; another pierces the mylohyoideus and anastomoses with the submental branch [2]. In 80–90% of the cases, sublingual arteries penetrate lingual cortical plate of the mandible through lingual foramen anastomosing with* incisive arteries* [3, 4]. These ones are terminal branches of inferior alveolar arteries, run in the incisive canals, supply blood to the anterior mandible, frontal teeth, and gingiva, and anastomose with sublingual arteries.

Severe haemorrhage from this anastomosing plexus has been reported as a complication of dental and surgical procedures involving perforation of the lingual cortex and/or laceration of the adjacent soft tissues [5]. In our patient the cause of the haematoma was the perforation of the lingual cortex caused by the bur during the placement of four postextractive osseointegrated implants in anterior mandible using flapless technique. Severe bleeding in the floor of the mouth with large-size haematoma involving sublingual, submandibular, and submental spaces causes swelling of the floor of the mouth and elevation and posterior displacement of the tongue with upper (oropharyngeal) airway obstruction similar to Ludwig's angina. Security airway patency should be the first management step in these situations using Guedel pattern airway. If the patient is able to breathe autonomously with good oxygen saturation value, intubation or tracheostomy is not necessary. Local bleeding control is very important, applying bimanual compression downward from floor of the mouth and lingual surface of the mandible and upward from submental skin. In addition, the drilled holes, when empty, should be filled with haemostatic agents such as fibrin sponge and oxide cellulose. Surgical incisional drainage is not indicated because it can facilitate the ulterior bleeding and limit the self-tamponading action by haematoma itself [6]. Nevertheless, if the bleeding fails to tamponade and turn into a dissecting haematoma, the main source of bleeding must be topographically identified through angiography, Angio-MRI, or CT scan images with contrast medium; sometimes the application of metal artefact correction algorithm in CT or the execution of MRI and CT many hours after the bleeding starts prevents obtaining high quality images. If the bleeding source is identified, the artery could be clipped through a full thickness mandibular flap of the lingual mucosa surface. Some authors recommend external ligation of the carotid artery or angiographic embolization if the bleeding cannot be controlled by a direct approach [7, 8].

Implant-based prosthetic rehabilitation in the anterior mandible is becoming a standard management option in partial or total edentulous patients. Flapless postextractive technique is one of the techniques used for implant placement in the intercanine area and is considered to be safe. However, severe and potential life-threatening complications, such as severe arterial bleeding and large-sized haematomas in the floor of the mouth, may occur. If, during the implant placement in the intercanine area, intense acute pain is noticed, haemorrhage and postdrilling progressive swelling, immediate suspension of surgical procedures, inside-outside bimanual compression on the floor of the mouth, Guedel pattern airway insertion, and local (haemostatic agent) and systemic (cortisone and tranexamic acid) medical therapy should be carried out. The prevention of these complications is mandatory with clinical and CT analyses, in order to highlight mandibular atrophy and to select carefully the correct length and angulation of the bone drill and to keep more attention to the risks linked to the flapless technique choosing the elevation of a lingual mucoperiosteal flap to access the mandibular contour intraoperatively and to protect the sublingual soft tissues and vasculature in risky cases.
